# Characterization of Rough PTFE Surfaces by the Modified Wilhelmy Balance Technique

**DOI:** 10.3390/polym12071528

**Published:** 2020-07-10

**Authors:** Christian W. Karl, Andrey E. Krauklis, Andrej Lang, Ulrich Giese

**Affiliations:** 1 Materials and Nanotechnology Dept., SINTEF Industry, Forskningsveien 1, 0373 Oslo, Norway; andykrauklis@gmail.com; 2 German Institute of Rubber Technology (DIK e. V.), Eupener Str. 33, 30519 Hanover, Germany; andrej.lang@dikautschuk.de (A.L.); ulrich.giese@dikautschuk.de (U.G.)

**Keywords:** polytetrafluoroethylene, PTFE, roughness, fractal dimension, mean roughness, contact angle, contact angle hysteresis, wetting, white light interferometry, modified Wilhelmy balance technique

## Abstract

The wetting of rough polymer surfaces is of great importance for many technical applications. In this paper, we demonstrate the relationship between the mean roughness values and the fractal dimension of rough and self-affine PTFE surfaces. We have used white light interferometry measurements to obtain information about the complex topography of the technical surfaces having different height distributions. Two different methods for the calculation of the fractal dimension were used: The height difference correlation function (HDC) and the cube counting method. It was demonstrated that the mean roughness value (*R_a_*) correlates better with the fractal dimension *D_f_* determined by the cube counting method than with the *D_f_* values obtained from HDC calculations. However, the HDC values show a stronger dependency by changing the surface roughness. The advancing and receding contact angles as well as the contact angle hysteresis of PTFE samples of different roughness were studied by the modified Wilhelmy balance technique using deionized water as a liquid. The modified Wilhelmy balance technique enables the possibility for future analysis of very rough PTFE surfaces which are difficult to investigate with the sessile drop method.

## 1. Introduction

The lotus effect, which describes the low wettability of a surface, is an important example for the wetting of super-hydrophobic surfaces. This is due to the surface microstructuring and the hydrophobic properties of epicuticular waxes on the leaf surface [1,2]. Water rolls off in drops and takes all dirt particles on the surface of the lotus leaf with it. There are various technical applications of hydrophobic, dirt-repellent surfaces such as self-cleaning roofing tiles, paintings, profiles as well as icephobic coatings for the prevention of ice accumulation [3,4,5].

The wettability of a surface can be tailored by the chemical composition of the surface and the degree of surface roughness [6]. The surface wettability is typically characterized by the contact angle, which represents the shape of the testing liquid on the solid. Contact angle measurements are the most surface sensitive of any common analysis technique having an analysis depth of ca. 0.5–1 nm [7]. The roughness induced wetting is widely discussed in the literature.

In previous investigations, it was shown that if the diameter of the drop is three orders of magnitude larger than the scale of mean roughness value (*R_a_*) of the investigated surface the roughness does not affect the contact angle [8,9].

**Figure 1:**
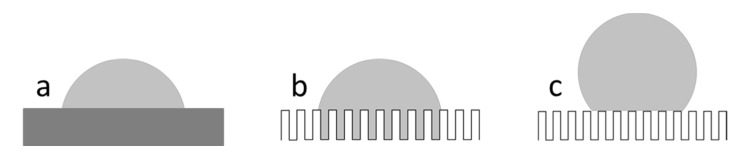
Representation of different wetting regimes: (**a**) Flat substrate; (**b**) Wenzel state; and (**c**) Cassie-Baxter state.

Contact angles of real surfaces, in contrast to ideal surfaces according to Young [10], are described by the roughness of the surface. In general, two types of wetting states are observed besides wetting on a flat substrate (see Figure 1a) on rough surfaces: The Wenzel and the Cassie state. As far as the Wenzel state is concerned, the surface grooves are filled by the water drop (see Figure 1b). This leads to the pinning of the drop to the surface. The wetting liquid penetrates completely into the depressions of the rough surface, which is called homogeneous wetting. In the case of heterogeneous wetting of a rough and chemically homogeneous surface, the so-called Cassie state (see Figure 1c), the drop does not penetrate the rough surface due to the entrapment of air. The resulting contact angle is larger than in the case of the Wenzel model because the interface between the two substances is smaller. Solid surfaces are divided into four categories. If the contact angle is less than 10 degrees, then the surfaces are superhydrophilic. Hydrophilic surfaces have contact angle values between 10 and 90 degrees. Contact angle values between 90 and 150 degrees are known as hydrophobic surfaces. Superhydrophobic surfaces such as the lotus leaf with its self-cleaning properties have contact angle values above 150 degrees or have a low tilting angle of 10 degrees [11,12]. Superhydrophobicity of surfaces can be adjusted by choosing an appropriate morphology or surface texture. In this way, a superhydrophobic surface is obtained instead of a hydrophobic one. The surface morphology can have micro-and/or nanoscale textures [13]. The lotus leaf with its hierarchical structure consisting of nanoscale wax protrusions on microscale roughness exhibits superhydrophobic properties having a stable Cassie state. Air is trapped in the cavities and as a result the Cassie-Baxter state is stabilized, which produces superhydrophobicity [14].

Different techniques to produce artificial superhydrophobic surfaces based on hierarchical structures have been studied. Especially, the fabrication of the rough structures with polymers as substrates are described in the literature [15,16,17].

In contrast to ideal surfaces, real surfaces (see Figure 1a) cannot be characterized by a single stable macroscopic contact angle, which is called the apparent contact angle. Consequently, there are different macroscopic contact angles [18]. These angles, which are described by metastable states, are due to the locally different inclination of the topography and thus correspond to several local minima of the free enthalpy of a liquid drop on a solid surface. Energy barriers exist between these minima. In an energetic equilibrium, where the Gibbs energy has the lowest value, the system is in its most stable state. The corresponding most stable macroscopic contact angle is called θ_eq_ [19,20]. It is calculated from the mean of the advancing and receding contact angles [18,21]. As a prerequisite for measuring the contact angle according to Marmur, a ratio between the drop diameter and the lateral extension of the roughness structures of at least three orders of magnitude is required [18].

Contact angle hysteresis, which is the difference between an advancing and receding contact angle has been investigated and discussed in the literature for a long time. However, the underlying mechanisms are still controversial. Possible causes are the surface roughness [22,23], the chemical heterogeneity of the surface [24,25,26], and time-dependent interactions of a solid with a liquid interface, resulting in swelling, liquid penetration into the surface area, and reorientation of the surface of functional groups [27,28]. Extrand and Kumagai stated that the range of the contact angle hysteresis was mostly a property of the system liquid-polymer [29,30].

The system polytetrafluoroethylene (PTFE)/water has been investigated by the dynamic sessile drop and tilting angle methods in several studies. Schulze et al. have received the hysteresis-free contact angle value, which is considered as a thermodynamic equilibrium contact angle from sessile drop measurements on different rough PTFE surfaces [31]. Other authors such as Extrand and In Moon investigated the contact angle on flattened PTFE surfaces and PTFE spheres [32]. Ruiz-Cabello et al. studied smooth PTFE surfaces (*R_a_* < 0.1 µm) and found a general disagreement between the sessile drop and the captive bubble methods [33]. Pericet-Camara et al. investigated PTFE surfaces by the tilting plate technique and the sessile drop method. As far as the tilting angle drop method is concerned, the sessile drop platform is inclined in steps of a 0.5° tilting angle with respect to the horizontal plane. Gravity moves the drop downwards in the inclined plane at the upper side. The drop is brought into an asymmetrical shape and only moves when the drop has reached a certain size. The advancing contact angle is the angle at the bottom, the angle at the top is the receding contact angle. They obtained contact angle hysteresis values with high values between 40–60° [34].

Contact angle goniometry is a powerful technique to observe the contact angle between the tangent to the liquid-gas and liquid-solid interfaces at the three-phase contact line. This method is widely used as a screening experiment for smooth surfaces [35]. To estimate the contact angle from the drop profile different methods are used: Spherical cap approximation [36], polynomial fitting [37], tangent line or Young-Laplace equation [38]. It was shown that different algorithms give different values of contact angles [39,40].

The contact angle goniometry is not very accurate for rough and hydrophobic surfaces, because the contact point between the axial location of the base line and the projected droplet boundary can appear distorted [41]. There are substantial inaccuracies as far as image processing is concerned, especially for surfaces which are superhydrophobic. Optical errors lead to systematic errors with respect to the determination of the droplet shape and tangent line [42]. It is difficult to determine the location of the baseline. The deviations of the measured contact angles can be large [43]. Contact angle measurements using the sessile drop technique depend on the experience and skills of the user. There are large deviations even if an experienced user performs the measurements [44]. Vuckovac et al. have shown that errors increase for superhydrophobic surfaces. The increase of the image resolution can be reduced slightly [42]. However, Heib and Schmitt have developed the so-called high-precision drop shape analysis (HPDSA), which involves a transformation of images from sessile drop experiments to calculate physically meaningful contact angles and to improve the disadvantages of the sessile drop goniometer method [45].

In comparison with the contact angle goniometry, the Wilhelmy balance technique has many advantages. The method is fully automated, and the influence of the experimenter is significantly reduced. Furthermore, it has a precise definition of the kinetic stages of advancing and receding and is efficient in the measurement of advancing and receding states (e.g., immersion rate) [46]. Recently, it was shown that the modified Wilhelmy balance technique can also be used for irregular shaped specimens instead of regular shaped samples having a constant perimeter [47]. In previous works, the contact angle hysteresis of elastomers was correlated with roughness factors which were obtained from white light interferometry measurements [19,48]. Little work has been done to correlate contact angles and contact angle hysteresis values of PTFE with roughness parameters, such as fractal dimension and height profile data [49,50,51]. To address this issue, we used various smooth and rough PTFE surfaces to investigate the influence of roughness parameters similar to the mean value *R_a_* and surface descriptors such as the fractal dimension *D_f_* on the contact angle and contact angle hysteresis. The surface descriptors such as the fractal dimension was calculated from the white light interferometry data. For this purpose, the roughness length (height difference correlation (HDC) function) and the cube counting methods were used to calculate the fractal dimension *D_f_* and the surface descriptors.

## 2. Materials and Methods

A polytetrafluoroethylene TECAFLON PTFE naturally produced by Ensinger GmbH, Nufringen, Germany was investigated in this study. The PTFE test specimens (length: 3 cm, width: 1 cm, thickness: 2 mm) were covered on both sides with different types of SiC sandpaper using Matador Nassschleifpapier P60-ST7000 (Starcke GmbH & Co. KG, Melle, Germany) and pressed for 3 min at 30 bars between two polished press plates using the vulcanization press WLP63/3.5/3 (Wickert Maschinenbau GmbH, Landau in der Pfalz, Germany) at a temperature of 25 °C. The samples were subsequently cleaned with 2-propanol (p. a., Merck) in the ultrasonic bath Sonorex Super (Bandelin electronic GmbH & Co. KG, Berlin, Germany) for 2 hours at (23 ± 1) °C. Different grits were used (2000, 1000, 400, 240, 60, and 30) which correspond to the coarseness of the abrasive particles or coarseness of the surface. It is a dimensionless number; the larger this number is to be considered, the smaller is the diameter of the grinding grains (grain size). This corresponds to the grain sizes 10.3, 18.3, 35, 58.5, 269, and 642 according to ISO 6344-2 and 3:1998 [52,53]. Additionally, unmodified PTFE specimens were used as reference material (denoted as unmodified).

A white light interferometer (“FRT-CWL 300”, lateral resolution: <2 μm; height resolution: 10 nm) from FRT (Fries Research & Technology GmbH, Bergisch Gladbach, Germany) was used to investigate the topography of the seven different PTFE surfaces and to obtain the height profiles of the surfaces. An area of 4 mm^2^ was measured with 1000 × 1000 measuring points per sample. In addition, an area of 64 mm^2^ was examined for the very rough surfaces with the grits 30 and 60.

The measured raw data for the representation of the topography were considered using the software program “Igor Pro” after deduction of the plane. “Igor Pro” was also used to calculate the surface descriptors, the fractal dimension, as well as the mean roughness (*R_a_*) (according to DIN EN ISO 4287) [54]. Fractal dimensions were calculated also using the cube counting method in the software Gwyddion (GPL, Brno Czech Republic).

**Figure 2:**
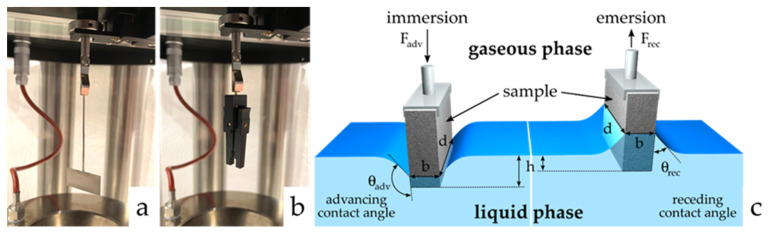
DIN-standardized platinum plate for the determination of the surface tension of the liquid (**a**), precision clamp as a sample holder for the polytetrafluoroethylene (PTFE) specimen (**b**) and schematic representation of the immersion and the emersion cycles of the PTFE samples for the determination of advancing and receding contact angles of the modified Wilhelmy balance technique (**c**).

For the conventional Wilhelmy method, by which the surface tension of the liquids is determined, a rectangular, DIN-standardized, and roughened platinum plate PT 11 (DataPhysics Instruments GmbH, Filderstadt, Germany) is applied (see Figure 2a) [55]. We have used the DCAT 11 Dynamic Contact Angle Tensiometer (DataPhysics Instruments GmbH, Filderstadt, Germany) for our investigations. A precision clamp PSH 11 (DataPhysics Instruments GmbH, Filderstadt, Germany) with defined dimensions, which is available for the DCAT 11 system was used instead of a platinum plate (see Figure 2b). Figure 2c reveals the immersion and emersion cycles of the PTFE samples for the determination of advancing and receding contact angles. The specimens were first cleaned for 2 h in an ultrasonic bath using 2-propanol as liquid and after the fabrication process the samples were cleaned with deionized water (DI) and dried before use at room temperature (23 ± 1) °C. Before dipping the sample in a test liquid, the surface tension of DI water was determined by the Wilhelmy Pt-Ir-Plate. The surface tension of DI was 72.7 mN m^−1^ at (23 ± 1) °C.

First, the sample is attached to the sample holder which is shown in Figure 2b. The holder with the attached sample is mounted on the force sensor holder of the tensiometer. After balancing the weight force (mg=0), the test specimen is immersed in the water and emerged with a scan rate of 0.1 mm/min. The technical design of the device ensures that the weight force was balanced. The accuracy of the balance is ±100 μg. The forces *F_adv_* and *F_rec_* (s. Equations (1) and (2)) are measured as a function of the immersion depth *h*. V=h·b·d is the volume and l=2·(b+d) is the wetted length of the test specimen, and γ_lv_ designates the surface tension and $\rho_{lv}$ the density of the solvent.

(1) $$F_{adv}=l\gamma_{lv}cos\theta_{adv}-V\rho_{lv}g+mg$$

(2) $$F_{rec}=l\gamma_{lv}cos\theta_{rec}-V\rho_{lv}g+mg$$

By linear regression to the immersion depth zero, the buoyancy force $F_{a}=V\cdot \rho \cdot g$ can be eliminated from the recorded force-distance diagrams. If the sum of buoyancy and weight force is equal to zero, the resulting force corresponds to the wetting force. Hence, the corresponding measured forces *F_adv_* and *F_rec_*_,_ from which the contact angles $\theta_{adv}$ and $\theta_{rec}$ can be calculated are obtained separately for the extrapolation of h to 0 (i.e., V=0) [56].

(3) $$\theta_{adv/rec}=arccos\left(\frac{F_{adv/rec}\left(h=0\right)}{l\gamma_{lv}}\right)$$

## 3. Results

In the following, the results of the white light interferometry measurements and the wetting investigations will be shown. First, the topography and height distributions of the PTFE surfaces will be described (Section 3.1). Subsequently, the surface descriptors such as the fractal dimension of the PTFE surfaces are calculated from the height difference correlation (HDC) function (Section 3.2). Since the fractal dimension *D_f_* is an arguably important parameter in the description of fractal rough surfaces, the box counting method, more precisely the “cube counting” method, is used in addition to the HDC (Section 3.3). The results of the correlation of *D_f_* with the grit number and *R_a_* and the determination of contact angles by the modified Wilhelmy balance technique are revealed in Section 3.4 and Section 3.5. The results of the determination of the equilibrium contact angle from rough PTFE surfaces are explained in Section 3.6.

### 3.1. Topography and Height Distributions of the PTFE Surfaces

**Figure 3:**
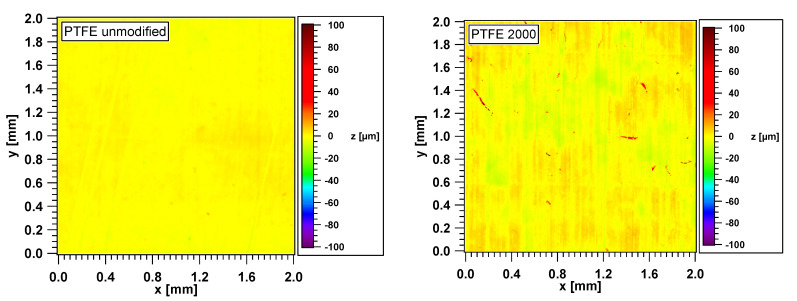

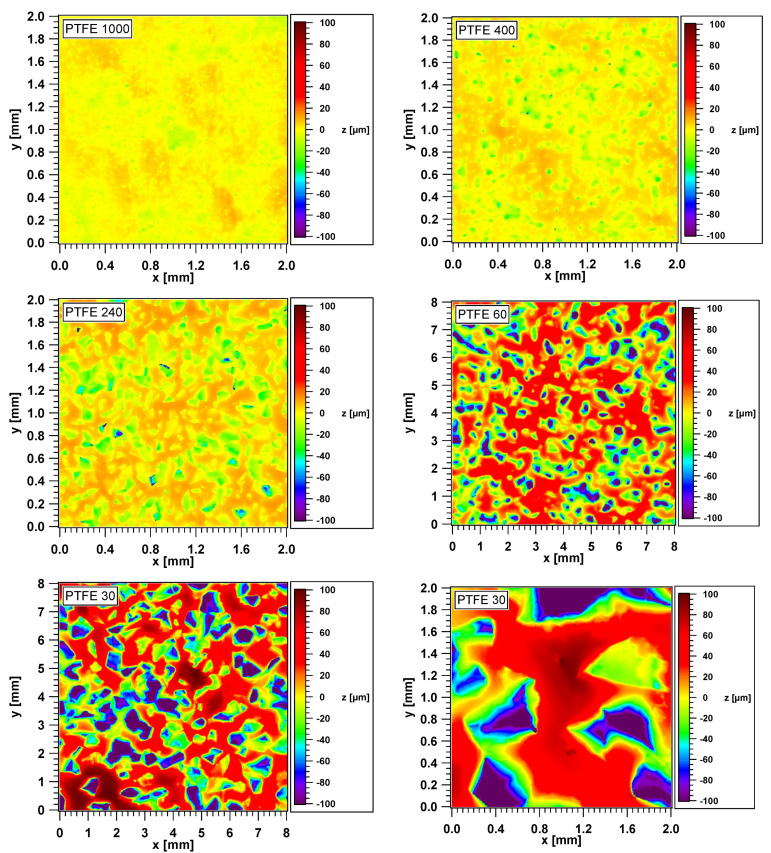
White light interferometric micrographs of the different smooth and rough PTFE samples (side B).

The modification of the PTFE surfaces by using SiC sandpapers with different grain sizes reveals as a change of the surface topography with different roughness. Therefore, each topography is a counterprint of the respective sandpaper surface. Figure 3 shows the white light interferometric micrographs of the seven different smooth and rough PTFE sample topographies (side B, see Table 1) which were used.

One backside of each of the three PTFE samples is shown for a certain roughness, which results from the different grain sizes (see Section 2). To consider as many structural details of the surfaces used as possible, a scanning area of 2 × 2 mm^2^ was considered. For the sake of a clearer representation, the samples with the grits 30 and 60 were shown with a scan size of 8 × 8 mm^2^. Additionally, the roughest sample with the grit 30 (particle size of the grains 642 micrometers) is shown in the scan sizes 2 × 2 and 8 × 8 mm^2^ for a better comparison with the other samples. Some of the PTFE samples differ very significantly in terms of roughness. The untreated sample used as a reference shows only very small differences in height. These are deviations of ca. ±5 µm, whereas the sample with the grit 2000 exhibits height differences of ca. ±15 µm. The samples with the grits 1000, 400, and 240, corresponding to the grain sizes 18.3, 35.0, and 58.5 micrometers, show height differences of ca. ±15, ±20–25, and ±50–60 µm, respectively. The samples with the grits 30 and 60 both show similar height differences of about ±85–95 µm. The height differences increase with the increasing grain size and decreasing grit. The grains of the abrasive paper dig deep into the PTFE surface and form partly narrow but deep holes. 

**Figure 4:**
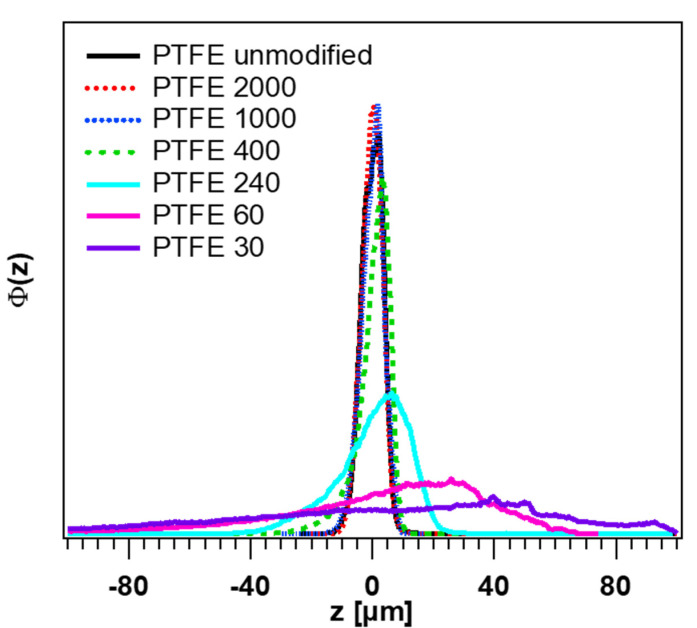
Distributions of the PTFE surfaces (side B) obtained by white light interferometry.

In Figure 4, the height distributions of the PTFE surfaces obtained by white light interferometry are revealed. The untreated PTFE surface shows very similar narrow height distributions as the two samples with the grits 1000 and 2000. The height distributions of the samples with the grits 240 and 400 are somewhat wider, whereas the sample with the grit 240 has a Gaussian-like distribution. The samples with the grits 30 and 60 have partly very narrow and deep holes with very different diameters, therefore the height distributions are very wide, especially in the sample with the smallest grit 30.

### 3.2. Height Difference Correlation Function and Fractal Descriptors of the PTFE Surfaces

In general, randomly rough surfaces such as sandpaper show stochastically self-affine structures, i.e., the topography is statistically invariant under anisotropic dilations. This so-called fractal nature of different surfaces can be mathematically determined by the height difference correlation function $C_{z}\left(\lambda \right)$.

(4) $$C_{z}\left(\lambda \right)={\langle (z\left(x+\lambda \right)-z\left(x\right))}^{2}\rangle$$

**Figure 5:**
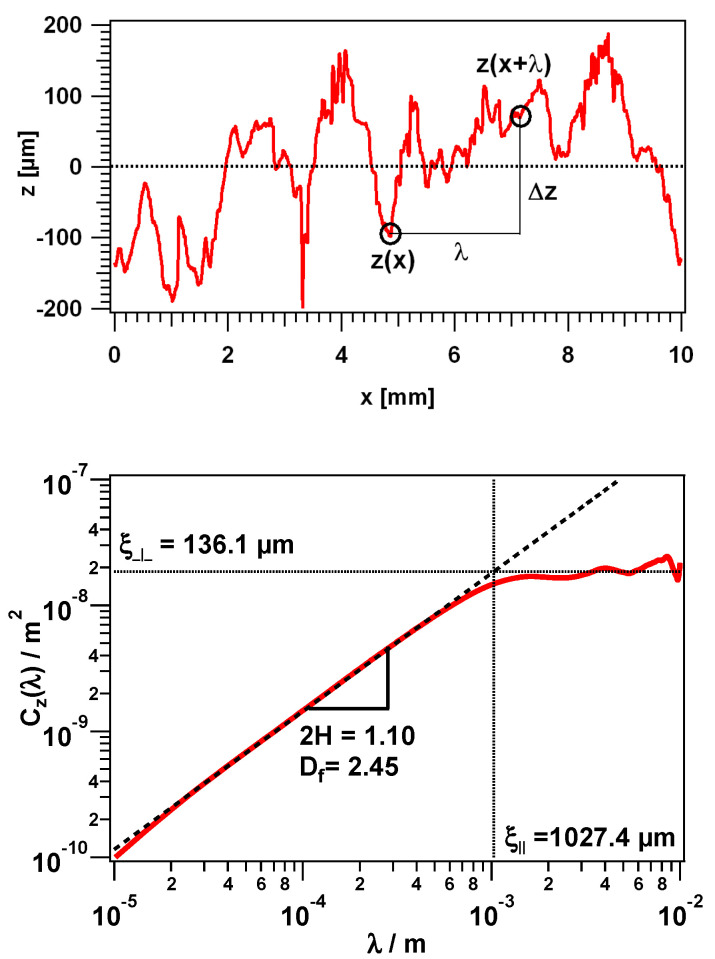
Measured profile of a rough granite surface (**top**) and the resulting height difference correlation function (**bottom**) with the surface descriptors $\xi_{∥}$, $\xi_{⊥}$, and $D_{f}$.

This function describes the mean square height differences ${\langle (\Delta z)}^{2}\rangle ={\langle (z\left(x+\lambda \right)-z\left(x\right))}^{2}\rangle \;$ of all roughness values, which are separated laterally by the length scale λ=Δx. An evaluation example for a rough granite surface is shown in Figure 5. With increasing lateral separation between the measured points, the height difference is increasing, the slope is given by the Hurst exponent H, which determines the fractal dimension of the surface ($D_{f}=3-H$). The fractal nature implies that the topography has even finer structures on all length scales [57,58]. For real surfaces, however, there are limits within which a self-similarity is valid. In other words, there is no surface that behaves self-affine over an infinite number of length scales and thus has a finite range of wavelengths [59]. For smaller lengths, the limit is on the atomic scale, whereas for larger length scales, the self-affinity is limited by two lengths, which are characteristic of each surface. In the lateral direction, this is determined by $\xi_{∥}\;$ and in the vertical direction by $\xi_{⊥}$.

Below both these cut offs $\xi_{∥}\;$ and $\xi_{⊥}$, the roughness behavior can be simply approximated by the following expression:

(5) $$C_{z}\left(\lambda \right)=\xi_{⊥}^{2}{\left(\frac{\lambda }{\xi_{∥}}\right)}^{2\left(3-D_{f}\right)}\;\;for\;\;\;\lambda <\xi_{∥}$$

Consequently, the statistical description of a random rough surface can be realized by only three descriptors, the lateral cut off $\xi_{∥}\;$, the vertical cut off $\xi_{⊥}$, and the fractal dimension $D_{f}$.

**Figure 6:**
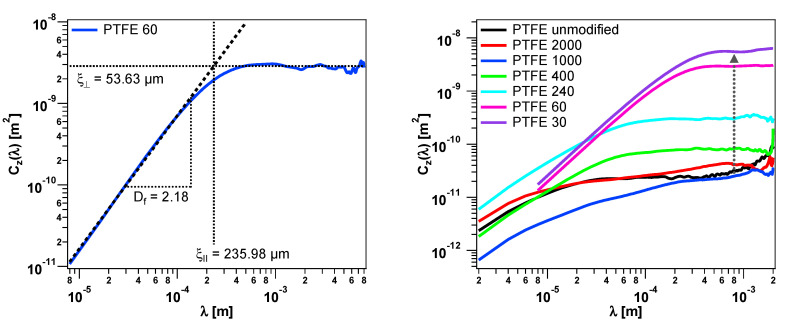
Height difference correlation functions $C_{z}\left(\lambda \right)$ of the PTFE samples (side A) with the grain size 60 fitted with one scaling regime. The obtained surface descriptors are indicated (**left**). Comparison of selected height difference correlations (**right**).

By applying this method on the PTFE surfaces, the differences can be quantified. In Figure 6 (left), an example for the sample PTFE 60 (side A) is shown, which reveals an expected curve. With increasing lateral separation, the height difference increases too. In this case, the surface roughness can be statistically characterized by $\xi_{∥}=236\;µm\;$, $\xi_{⊥}=54\;µm$, and $D_{f}=2.18$. Values for all the PTFE samples can be found in the front (side A) and the back (side B) of one specimen of the three samples for each kind of sample were evaluated.

By comparing all the PTFE samples, a systematic trend can be observed. With the increasing grain size of the counter printed sandpaper, the vertical cut off is increasing due to higher roughness, which can be well seen in the broadness of the height distribution (see Figure 4). This is clear, because the vertical cut off length is closely connected with the variance $\tilde{\sigma }$ of the surface roughness by $\xi_{⊥}=\tilde{\sigma }\cdot \sqrt{2}$ [60]. Another observation is the shift of the lateral cut of length $\xi_{∥}$ to higher values. It means that the statistical repeatability of the surface depends on the lateral length scale. To take larger structures into account, a larger area must be observed. However, one thing seems to be the same for all samples. For smaller length scales, all curves approximate to a straight line in the double-logarithmic plot. From this, it can be deduced that the structure on small length scales is determined by the used material itself.

### 3.3. Cube Counting Method and Calculation of Fractal Descriptors of the PTFE Surfaces

**Figure 7:**
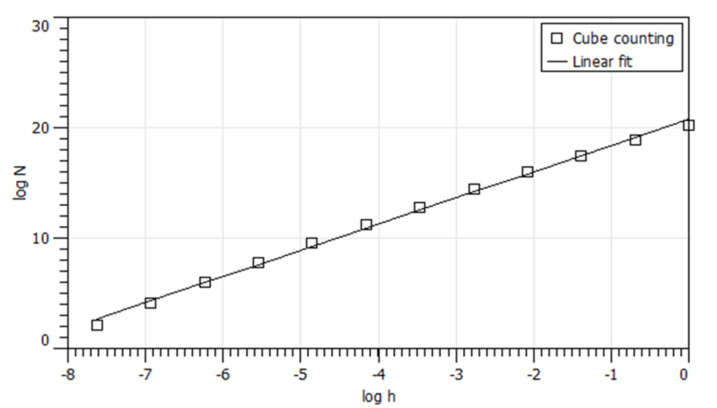
Log(N(l)) versus log(1/l) with h = 1/l of the sample with the grit 240 (side A) to determine the fractal dimension *D_f_* from the slope of the fit curve.

Objects that show random properties are often encountered. It can be assumed that these objects have self-affine properties within a certain scale range. Therefore, it can also be assumed that rough surfaces belong to the described random objects that have self-affinity. In earlier works, different surfaces have been investigated by atomic force microscopy (AFM) and scanning electron microscopy (SEM) pictures and characterized by fractal dimension values using the box counting method [61,62]. In the literature, different experimental methods are used to investigate these surfaces. For example, Zhang and Jackson have used a profilometer to characterize the profiles of surfaces of different roughness and to calculate the fractal dimension using various methods, including the cube counting method [63,64]. We also used the cube counting method to calculate the fractal dimension and compare it with the *D_f_* values from the HDC investigations. The cube counting method is derived from the box counting method. It is based on the fact that a cubic grid with the grid constant *l* is superimposed on the z-expanded area. At the beginning *l* is set to X/2, where X is the length of the edge of the surface. This leads to a grid of 2 × 2 × 2, which is a total of eight cubes. Hence, N(l) is the number of all cubes containing at least one pixel of the cube. In the next step, the grid constant *l* is reduced step by step by a factor of 2. This process is repeated until *l* is equal to the distance between two adjacent pixels [62,65]. The fractal dimension *D_f_* is then obtained from the slope of a plot of log(N(l)) versus log(1/l). This is shown in Figure 7 using the example of the sample with grain size 240. The obtained value for *D_f_* is 2.375.

### 3.4. The Relationship between the Fractal Dimension D_f_ and the Grit Number as Well as the Mean Roughness R_a_

**Figure 8:**
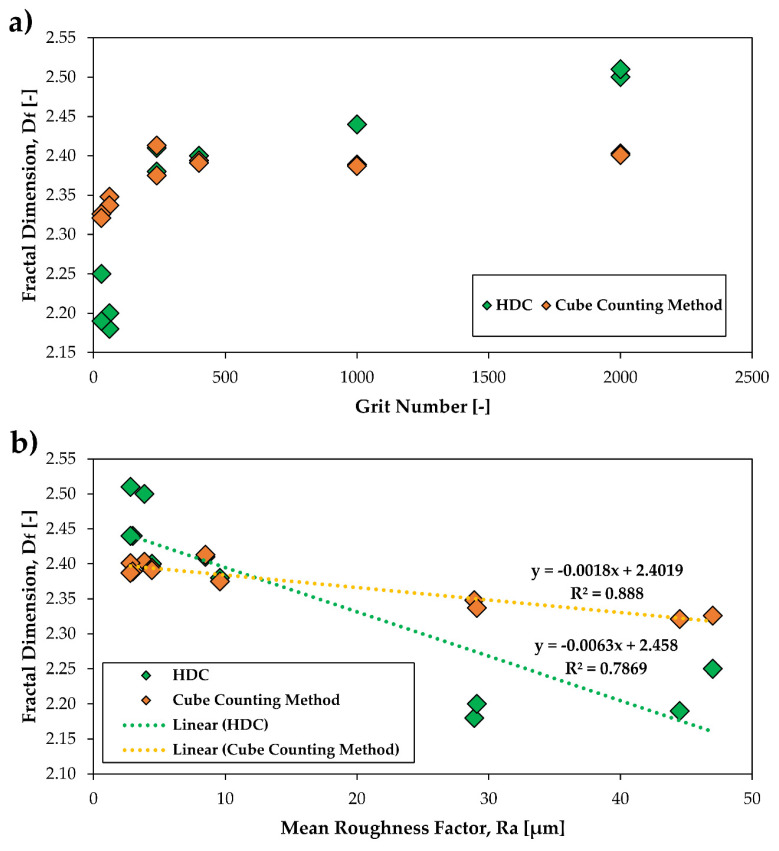
The fractal dimension *D_f_* calculated by the height difference correlation (HDC) and cube counting method (sides A and B) in dependence of the grain size number (**a**) and depending on the mean roughness (**b**).

Figure 8 on the left shows the fractal dimension as a function of the grit number for both sides A and B for one of the three samples (see Table 1). With the increasing grit number, i.e., with the decreasing grit size, the fractal dimension approaches a plateau value of approx. *D_f_* = 2.4–2.5 for the values calculated by HDC as well as by the cube counting method. The *D_f_* values calculated by the HDC function show slightly lower values for the small grit numbers 30 and 60 and slightly higher values for the grit numbers 1000 and 2000 than those obtained by the cube counting method. The unmodified PTFE sample has *D_f_* values of ca. 2.3 for the cube counting method. The HDC method exhibits higher values of approx. 2.5–2.6 for the unmodified PTFE surface. Zhang and Jackson investigated different rough surfaces and compared several methods for the calculation of the fractal dimension [63]. They also applied the roughness length method to which the height difference correlation function belongs. The roughness length method is widely used and gives good results as Klueppel and Zhang have shown previously [60,63]. Comparing the two methods, it was shown that the cube counting method follows more a linear trend as it was also verified for our results. Possibly, with the cube counting method, the scale ranges are represented differently.

Figure 8 on the right shows the dependence of the fractal dimension *D_f_* on the mean roughness *R_a_* of the used PTFE surfaces with different grits for both sides A and B of one specimen (see Table 1). The unmodified sample is not shown here because its grit number is not known. The fractal dimension is regarded as a measure of roughness. For smooth surfaces, the *D_f_* is 2.0, which gradually increases as the surface roughness increases. For very rough surfaces, *D_f_* stands very close to 3.0 [66]. As can be seen from the results, the mean roughness *R_a_* for our rough PTFE samples correlates better with the fractal dimension *D_f_* in the case of the cube counting method than in the HDC calculation. Especially, the samples with the grits 30 and 60 are very rough and they have mean roughness values between about 30 and 45 micrometers. The R_a_ values of these two rough samples correlate worse with the *D_f_* values calculated by the HDC method and their *D_f_* values are lower than those calculated by the cube counting method. However, the fractal dimension values calculated with the HDC method show a stronger dependency for different roughness values. It should be pointed out, that the cube counting method describes self-affine surfaces on average better whereas the HDC method in this work considers mainly small length scales.

### 3.5. Determination of Contact Angles by the Modified Wilhelmy Balance Technique

**Figure 9:**
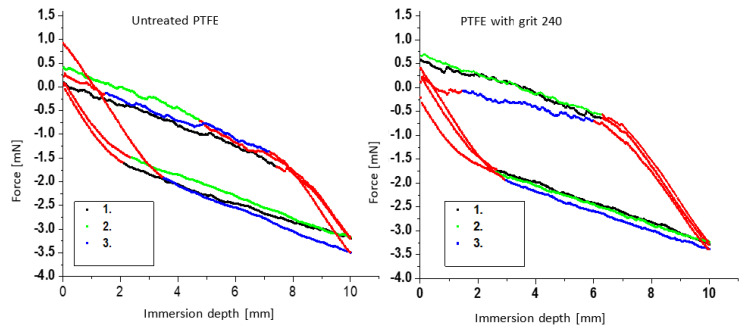
Representation of the first immersion and emersion cycles of three untreated (**left**, [67]) and roughened (**right**) PTFE samples (grit size 240) in water (areas shown in red: Not used to determine the force values).

To determine the dynamic contact angles and calculate the contact angle hysteresis, the modified Wilhelmy balance technique was used. Investigations were carried out with the PTFE samples of different roughness (see Section 2andSection 3.1). The contact angles are calculated according to Equation (3) in Section 2. In the case of the untreated PTFE sample, only a slight unevenness in the range of a few µm is visible. By roughening the PTFE surface, height differences of up to 95 µm are obtained (see Figure 3). The immersed area of a Wilhelmy sample (approx. 6.8 cm^2^) is much larger compared to the surface of a sample that is investigated using the sessile drop method (approx. 300 mm^2^ for 30 drops with a diameter of 1.2 to 1.5 mm) [19]. This ensures that a sufficiently representative total surface is wetted by the liquid (water) to characterize particularly rough surfaces. As an example, Figure 9 shows the first three immersion and emersion cycles of the three untreated (a) and roughened (grit 240, b) PTFE specimens in water. The first cycles were used in each case, since parts of the sample surfaces have already been wetted with water when the samples are immersed for the second time. Hence, deviations during contact angle determination can be avoided. Furthermore, this method is also intended to consider the deviations in the roughness of the sample. The areas shown in red colour were not used to determine the force values (*R*^2^ ≥ 0.98 for the fitted data points).

**Figure 10:**
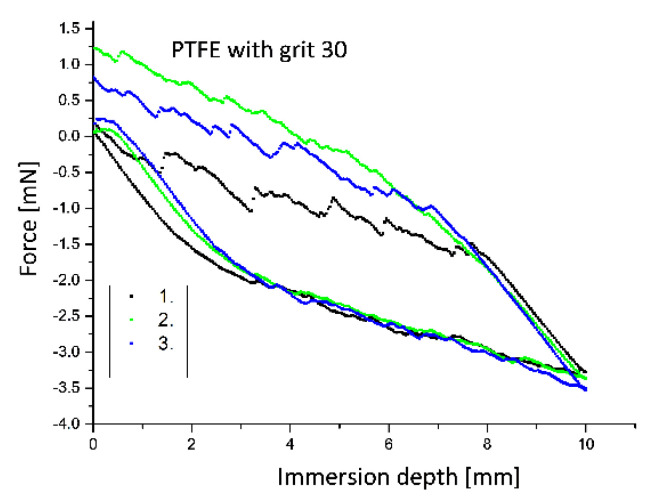
Representation of the first immersion and emersion cycles of the roughened PTFE samples (grit size 30) in water.

Figure 10 reveals the first three immersion and emersion water cycles of the roughened specimens with the grit 30. It is evident that the sample with the grit 240 covers a larger hysteresis area than the untreated sample. For the specimens with the grit 30 larger deviations can be observed. Furthermore, the course of the force values during immersion is no longer linear with the increasing roughness of the PTFE sample surface. Especially, with the rough PTFE surface with the grit 30, it is apparent that the force values during immersion are very similar and are well aligned on a straight line, whereas clear deviations are observed during emersion due to the rough surface structure (see Figure 9 and Figure 10). This is due to the fact that the specimen was not yet wetted when it was immersed and very small drops of water remain in the grooves when the specimen is removed. The larger deviations of the force values during the emersion process are due to the more irregular distribution of grooves in the PTFE surface of the rough sample with the grit 30 (see Figure 3 and Figure 4).

### 3.6. Determination of the Equilibrium Contact Angle of Rough PTFE Surfaces

**Figure 11:**
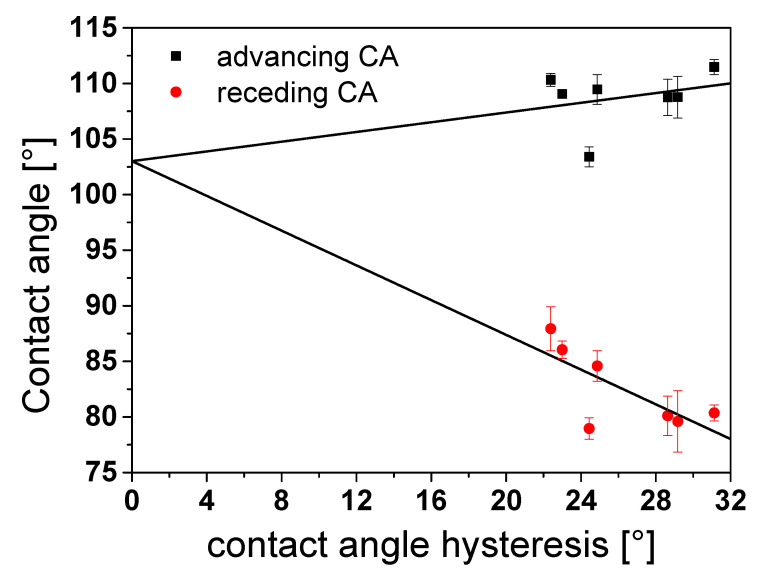
Contact angles as a function of the contact angle hysteresis for the system PTFE-water.

We used a plot from Kamusewitz with the advancing and receding contact angles versus the contact angle hysteresis to obtain the equilibrium contact angle. Based on the theory of Johnson and Dettre [68], Kamusewitz found an empirical relationship between the contact angle hysteresis and the theoretical parameter, the equilibrium contact angle. The two fitted lines of the advancing and receding contact angles versus the contact angle hysteresis intersect always exactly on the ordinate [31]. The point of intersection with the ordinate gives the so-called equilibrium contact angle, i.e., the contact angle in a thermodynamic equilibrium. This represents the ideal contact angle of a surface. Marmur and Volpe have described this as the most stable contact angle. This is related to the apparent contact angle associated with the lowest Gibbs energy state for a system and represents the global minimum energy [18,46]. In contrast to Kamusewitz et al. we did not perform sessile drop investigations, which can be more strongly influenced by the experimenter. Possibly, stick-slip effects occur also on PTFE surfaces using water as liquid. It was shown that these stick-slip effects take place during measurement on PTFE surfaces for different liquids and on other thermoplastic surfaces [69,70]. However, Orejon et al. have investigated PTFE surfaces which did not show significant pinning of the contact angle [71]. We used three specimens of one roughness respectively the grit number (e.g., grit 240, see Section 2) to consider the influence of sample variation. Our contact angle hysteresis values are between 22 and 32 degrees, whereas Kamusewitz’ values are in a hysteresis range of 20 to 70 degrees. Figure 11 shows our results for the previously described plot of the advancing and receding contact angle versus the contact angle hysteresis. The different rough PTFE sample surfaces shown in Figure 3 were used here. The deviation for the receding contact angles is larger than for the advancing contact angles since the surface of the specimen has already been wetted when it is emerged from the liquid. Furthermore, this deviation is also due to the different variations in roughness (see also Figure 4). The value for the equilibrium contact angle is 103 ± 8.5° (see Figure 11). Kamusewitz et al. obtained an equilibrium contact angle of ca. 95° [31]. This can probably be attributed to the fact that the deviations with the sessile drop method are larger. Therefore, it is difficult to compare the equilibrium contact angles of the sessile drop and Wilhelmy method. Additionally, the local inhomogeneities are responsible for the contact angle hysteresis. With the increasing roughness, evidence of the formation of air pockets on the specimens are found in the literature. As a result, the contact angle hysteresis values no longer increase or even become smaller. This was verified for the tilting and the goniometer sessile drop method [34]. The slightly larger deviations of the receding contact angles could also contribute to a higher equilibrium contact angle.

## 4. Discussion

The rough PTFE surfaces we used were comprehensively characterized using the height difference correlation function (HDC) and the cube counting method. We have also shown that the HDC is a good calculation tool to describe fractal descriptors such as the fractal dimension *D_f_* of rough polymer surfaces which are used for technical applications. There is a good correlation between the mean roughness value and the fractal dimension calculated from the cube counting method. Larger deviations have been obtained using the HDC method. We have demonstrated that the Kamusewitz plot is also suitable for higher contact angle hysteresis values with very rough samples with a broad size distribution and the calculation of the equilibrium contact angle is possible under these circumstances. The obtained equilibrium contact angle differs from the value obtained by the calculation from the advancing and receding contact angles of the sessile drop method described in previous works. This is due to the fact that the Wilhelmy method has a precise definition of the kinetic stages of advancing and receding and is efficient in the measurement of advancing and receding states (e.g., immersion rate for the sample with the grit 30, see Figure 11). Furthermore, the Wilhelmy method is fully automated in contrast to the sessile drop method. Hence, the influence of the experimenter is significantly reduced. We did not find a relationship between roughness and fractal dimension and contact angle hysteresis for our investigated roughness range.

## 5. Conclusions

Currently, the sessile drop method, especially in static mode, is often used to investigate the wetting of polymer surfaces. Moreover, the so-called sessile drop needle in the method, which also provides advancing and receding contact angles, has some other disadvantages, e.g., stick-slip effects, which can occur when measuring the contact angle. However, this method is not as well defined by the experimental procedure as the Wilhelmy method, so this dynamic method should be used to characterize polymers, especially for rough surfaces. Future works should focus on the relations of roughness and the chemical heterogeneity of the polymer surface on the contact angle and contact angle hysteresis to develop advanced models for the wetting of polymer surfaces. Our analysis has shown that the fractal dimension is a useful parameter for the characterization of rough technical surfaces. It would be promising for future works to look also at the relationship between contact angle hysteresis and fractal dimension of polymer surfaces having a different polarity.

## Figures and Tables

**Table 1:** Descriptors for the used sample pool. The front (side A) and the back (side B) of one sample were evaluated.

Sample (side)	$\xi_{⊥}$ [µm]	$\xi_{∥}$ [µm]	$D_{f}$ [-]
PTFE unmodified (A)	5.57	31.21	2.56
PTFE unmodified (B)	1.78	171.95	2.71
PTFE 2000 (A)	6.32	21.61	2.50
PTFE 2000 (B)	7.15	91.74	2.51
PTFE 1000 (A)	5.31	54.73	2.44
PTFE 1000 (B)	5.08	49.61	2.44
PTFE 400 (A)	8.86	44.74	2.40
PTFE 400 (B)	8.83	45.29	2.40
PTFE 240 (A)	17.88	48.39	2.38
PTFE 240 (B)	16.51	48.11	2.41
PTFE 60 (A)	53.63	235.98	2.18
PTFE 60 (B)	54.99	222.79	2.20
PTFE 30 (A)	77.95	425.35	2.25
PTFE 30 (B)	78.15	288.12	2.19
